# Incidence of stroke and its predictors among hypertensive patients in Felege Hiwot comprehensive specialized hospital, Bahir Dar, Ethiopia, a retrospective follow-up study

**DOI:** 10.1186/s40001-023-01192-6

**Published:** 2023-07-10

**Authors:** Solomon Misgana, Mulusew Andualem Asemahagn, Desta Debalkie Atnafu, Tadele Fentabil Anagaw

**Affiliations:** 1 Amhara Regional Health Beauro,Bahir Dar, Bahir Dar, Ethiopia; 2 School of Public health, College of Medicine and Health Science Bahir Dar University, Bahir Dar, Ethiopia; 3 Department of Health System Management and Health Economics, School of Public health, College of Medicine and Health Science Bahir Dar University, Bahir Dar, Ethiopia; 4 Department of Health Promotion and Behavioral Science, School of Public health, College of Medicine and Health Science Bahir Dar University, 079, Bahir Dar, Ethiopia

**Keywords:** Incidence, Stroke, Hypertension, Felege Hiwot referral hospital, Ethiopia

## Abstract

**Background:**

Globally, one in three adults has hypertension, a condition that causes 51% of all deaths from stroke. Stroke is becoming a major public health problem and the most common cause of morbidity and mortality among non-communicable diseases in the world and Ethiopia. Therefore, this study assesses the incidence of stroke and its predictors among hypertensive patients in Felege Hiwot Comprehensive Specialized Hospital, Bahir Dar, Ethiopia 2021.

**Methods:**

A hospital-based retrospective follow-up study design was used, simple random sampling technique was used to select 583 hypertensive patients that had follow-up registration between January 2018 and December 30th, 2020. Data were entered into Epi-data version 3.1 and exported to STATA version 14. The adjusted hazard ratio for each predictor with a 95% confidence interval was calculated using the Cox proportional hazards regression model, and a *P*-value ≤  0.05 was used to denote statistical significance.

**Results:**

From 583 hypertensive patients 106(18.18%) [95% CI 15–20] were developed stroke. The overall incidence rate was 1 per 100 person-years (95% CI 0.79–1.19). Comorbidities (Adjusted hazard ratio(AHR): 1.88, 95% CI 1.0–3.5), stage two hypertension (AHR = 5.21, 95%CI 2.75–9.8), uncontrolled systolic blood pressure (AHR: 2, 95% CI 1.21–354), uncontrolled diastolic blood pressure (AHR:1.9, 95% CI 1.1–3.57), alcohol consumption (AHR = 2.04, 95%CI 1.2–3.49), age 45–65 (AHR = 10.25, 95%CI 7.47–11.1); and drug discontinuation (AHR = 2.05,95% CI 1.26–3.35) were independent predictors for the incidence of stroke among hypertensive patients.

**Conclusion:**

The incidence of stroke among hypertensive patients was high and various modifiable and non-modifiable risk factors highly contributed to its incidence. This study recommends early screening of blood pressure, giving priority to comorbid patients and patients with advanced stage hypertension, and giving health education about behavioral risks and drug adherence.

## Introduction

The current world health organization definition of stroke is still similar with 1970 definition which is a rapidly developing clinical sign of focal or global disturbance of cerebral function waiting for greater than 24 h or results in death with no apparent cause other than that of vascular origin [1]. Ischemic and haemorrhagic strokes are common. Ischemic is due to lack of blood flow and haemorrhagic is due to intracranial bleeding. Ischemic accounts for about 85% of all strokes and others cover 15% [2, 3].

Risk factors for stroke are modifiable (Diabetes mellitus (DM), Hypertension (HTN), alcohol consumption, smoking, and obesity) and non-modifiable risk factors (Sex, age, race, and ethnicity) [4]. In Sub Sahara Africa (SSA) HTN is the commonest risk factor [5]. Based on the study conducted in Debre Markos Referral hospital HTN was the most common risk factor in 34.6% of stroke patients, [6]. Clinically, stroke is diagnosed according to WHO definition and by supporting medical instruments like CT scan and MRI [7].

In 2016, the prevalence and incidences of stroke cases amounted to nearly 83 million and 13.6 million, respectively [8]. The incidence and death rate is increasing in Low-and Middle-Income Countries particularly in Africa contributing 86% of deaths for the globe [9–11]. In Ethiopia about 24% of all neurological admissions are due to stroke [12, 13]. According to WHO data published in 2017, stroke deaths in Ethiopia reached 6.23% of total deaths [14]. In 2019, the overall in-hospital mortality rate of stroke was 18% (95% CI 14–22) [15].

Globally 1.13 billion people have HTN and in Ethiopia it affects 15.9% of the population (FMOH) [17]. High blood pressure (BP) puts an extra strain on the blood vessels which makes a stroke due to a clot (ischemic stroke) or by burst of blood vessels (haemorrhagic stroke) [18]. HTN needs special emphasis which increases the burden of stroke [19, 20]. Stroke is a global health problem and one of the leading causes of morbidity, mortality and disability [21]. WHO reports, 15 million people develop stroke [22]. In 2017, 6.2 million deaths were recorded [23]. Globally, it is the second leading cause of death, and physical disability [24]. In 2013, 113 million DALYs due to stroke were recorded [25].

Currently, stroke is one of the greatest public health problems, accounting for 7% of total deaths [32]. HTN is responsible for 66.2% of all stroke admission [33]. In 2015/16–2019/20, the Ethiopian HSDP projects to decrease premature mortality from NCD by 12.5% [34]. The world has experienced double burden of diseases by NCD and CD simultaneously including Ethiopia. Although admission of stroke patients to the hospitals due to HTN is increased from time to time, there are limited findings that aim to explore those factors. The overall in-hospital mortality rate of stroke was 18% (95%:14–22). Prevalence of stroke in FHCSH was 7.5% and the in hospital mortality in 2020 was 127 (16%) which covers the highest death rate compared to other causes of death. Therefore, this study aims to assess the incidences and factors associated with stroke among HTN patients at FHCSH, Bahir Dar, Ethiopia, 2021.

## Methods and materials

### Study area and period

The study was conducted at Felege Hiwot Comprehensive Specialized Hospital (FHCSH) that is found in Amhara region, Bahir Dar city at a distance of 560 KM from Addis Ababa, the capital city of Ethiopia [35]. In the hospital total 500 formal beds, 11 wards, 39 clinical and non-clinical departments providing Diagnostic, curative and Rehabilitation service at outpatient and inpatient-based services and serving over 7 million people from the surrounding area. From January 2018 up to December 2020, FHCSH gave services for 4560 and 2262 hypertensive and stroke patients. The actual data collection was carried out from March 15/2021 to April 15/2021 based on medical chart review of hypertensive patients enrolled in FHCSH NCD Unit from the January 1st of 2018 to the December 30th of 2020 time periods.

#### Study design

Hospital-based retrospective follow-up study design was applied.

#### Source population

All hypertensive patients in FHCSH.

#### Study population

Those hypertensive patients who had follow-up from 2018 to 2020 in FHCSH.

#### Inclusion criteria

All medical records of hypertensive patients in FHCRH during the defined period (2018–2020) were included.

#### Exclusion criteria

Patient’s chart that was missed at the time of data collection, charts not including information about the patient and charts of hypertensive mothers due to pregnancy were excluded from the study.

### Sample size determination

Sample size was calculated for both objective one and two using single and double population formula respectively. For first objective, a single population proportion formula for the outcome was used to calculate the sample size by considering the following statistical assumptions: *P *= Incidence proportion of stroke, based on hospital-based retrospective observational study was conducted in the medical ward of Debre Markos Referral Hospital from March 2017 to April 2019 the incidence proportion of stroke patients were 7.7% [6], Za/_2_ = z score of 95% CI and d = margin of error (5%). *n* = 110. Then after adding 10% contingency rate for non-respondents, the sample size will be 121. For the second objective, the sample size for associated factors was determined using the double population proportion formula, alcohol drink, and Diabetes Mellitus were significantly associated with survival time of hypertension patients [36]. Finally, the calculated sample size for independent factors was greater than the dependent variable. From the above listed predictors alcohol consumption gave the highest sample size. So, by considering non-response rate of 10% the final sample size was 583.

### Sampling procedure

At the beginning, all medical records of a confirmed diagnosis of hypertensive patients registered from January1st, 2018 to December 30th, 2020 were assessed. Then study participants were selected using simple random sampling techniques.

### Study variables

#### Dependent variable

Incidence of stroke.

#### Independent variables

*Socio-demographic* Age, sex, residence, family history of stroke, and ethnicity.

*Behavioural factors* Alcohol consumption and cigarette smoking.

*Comorbidities and physical measurement* Diabetes Mellitus, UTI, AKI, cardiac diseases, pneumonia, GCS and level of blood pressure.

*Treatment related factor* Antihypertensive drugs and drugs for stroke treatment.

### Case ascertainment and operational definition

#### Stroke

Rapidly developed clinical signs of focal or global disturbance of cerebral function lasting more than 24 h or leading to death with no apparent cause other than vascular origin [1].

#### Outcomes of stroke

Patients with reduced signs and symptoms as compared with the admission time will be categorized as improved. Those who will be directed or transferred from one health institution to another for better diagnosis and management will be described as referred. Patients who will be discharged from the hospital with advice and recommendations from the healthcare providers to promote their health will be categorized as “left against medical advice,” whereas, if the patient presents a dilemma for the physician to determine the outcome, he/she will be categorized as undetermined [6].

#### Uncontrolled and controlled blood pressure

Systolic blood pressure: controlled (< 140) and uncontrolled (> = 140), diastolic blood pressure: controlled (< 90) and uncontrolled (> = 90) [38].

#### Glasgow coma scale

GCS score of 13–15 (Mild brain injury), GCS score of 9–12 (Moderate brain injury), GCS score of 3–8 (Severe brain injury) [39].

#### Alcohol drinker

It refers to those participants who consume a drink containing alcohol [40].

#### Smoker

It refers to those participants who smoke any tobacco products (such as cigarettes, cigars, or rolled tobacco) [40].

#### Incidence proportion

Is the probability that a particular event, such as occurrence of a particular disease, has occurred before a given time [42].

#### Incidence rate

Is a measure of the frequency with which a disease or other incident occurs over a specified time period [42].

### Data collection methods

Structured questionnaire was used to collect the data. The questionnaire was adapted from WHO stroke assessment tool and previous studies [38, 43]. It was prepared in the English language and then translated into local language (Amharic language) by experts to check for consistency. The data were collected by health professionals who took training about data collection methods and procedures. The questionnaire involved closed-ended questions. The tool included socio-demographic factors, patient behaviour, comorbidities, and physical measurements and treatment related factors.

#### Quality assurance

Data quality was assured by designing appropriate data extraction tools. The questionnaire was translated to local language (Amharic) by professionals and back to English by another professional to see the consistency. Data were checked for completeness and consistency at the site of data collection. Training on data collection was given to data collectors and supervisors before data collection.

#### Data analysis methods

Data were entered into Epi-data 3.2 and exported to Stat version 14 for analysis. Descriptive statistics like frequencies and proportions were calculated to describe the study participants. The incidence density of stroke was calculated using person-years of follow-up as the denominator for the entire cohort. A Kaplan–Meier plot was used to estimate the probability of stroke-free survival. Bivariable Cox regression was fitted and those independent variables which fitted on the *P*-value less than or equal to 0.25 level of significance were included in the multivariable analysis [44]. The assumptions of the Cox proportional hazards regression model assessed by the global test, Schoenfeld residuals. Multiple Cox regression tests at *P*-value less than 0.05 were identified as predictors associated with incidence of stroke [45].The results of these models were expressed as adjusted hazard ratios (AHRs) with 95% confidence interval.

## Results

### socio-demographic characteristics of patients

All 583 hypertensive patient charts were reviewed with 100% response rate. Between January 1st 2018 and December 30th 2020 out of the 583 hypertensive patients, 477 were censored and 106 developed strokes. From study subject 51.5% were Male. Half of hypertensive patients were between the age of 45 and 65 years. The overall mean age was 58 ± 15.5 SD years (Table 1).

**Table 1:** Socio-demographic characteristics of hypertensive patients with their stroke status admitted to FHCSH, during January 2018 to Feb 2020, Bahir Dar. (*N* = 583)

Variables	Response	Stroke (No_-_, %)	Censored (No_._, %)	Total	Pearson chi2	*P*-value
Sex	Male	63 (20.8)	237 (79.2)	300 (51.5)	3.3	0.069
	Female	43 (15.3%)	240 (884.7)	283 (48.5)		
Age	< 45	1 (0.77)	128 (99.23)	129 (22.1)	44.5	0.001**
	45–65	78 (27.1)	210 (72.9)	288 (49.4)		
	> 65	27 (16.2)	139 (83.8)	166 (28.5)		
Ethnicity	Amhara	102 (17.8)	470 (82.2)	572 (98.1)	4.7	0.097
	Tigrie	0 (0)	2 (100)	2 (0.34)		
	Awi	0 (0)	0 (0)	0 (0)		
	Others^*^	4 (44.4)	5 (55.6)	9 (1.56)		
Residence	Rural	63 (25.4)	185 (74.6)	248 (42.5)		0.001**
	Urban	43 (12.8)	292 (87.2)	335 (57.5)	15.1	
Family history of stroke	Yes	104 (17.9)	477 (82.1)	581 (99.6)	9.0	0.003**
	No	2 (100)	0 (0)	2 (0.4)		
Smoking	Yes	58	2	60	276.9	0.001**
	No	48	475	523		
Alcohol drink	Yes	68	453	92	228.0	0.001**
	No	38	24	491		

^*^Other ethnicities like Afar, Benishangule, and Somali

**Factors those had *p*-value ≤ 0.05

### Duration of hospital stay and outcomes of stroke patients

Duration of hospital stays of stroke patients started with a minimum of 6 days to a maximum of 30 days and with a mean duration of hospital stay of 11 days ± 4.2 SD [95% CI 9.8, 11.4]. Among 106 stroke patients, 88.68% discharged with improvement and 1.89% of patients referred to other health institutions. Among all stroke patients 10 (9.43%) reported dead (Table 2).

**Table 2:** Duration of stroke patient’s hospital stay, stroke outcome and type at FHCSH, during January 2018 to Feb2020, Bahir Dar, Ethiopia. (*N* = 106)

Duration of hospital stay	Stroke outcome and type						Total
	Ischemic			Hemorrhagic			
	Dead	Improved	Referred	Dead	improved	Referred	
6 to 10 days	3	57	1	0	9	0	70
11 to 18 days	2	22	1	2	3	0	30
19 to 26 days	1	2	0	1	0	0	4
≥ 27	1	0	0	0	1	0	2
Total	7	81	2	3	13	0	106

### Stroke-free survival status of hypertensive patients

A total of 583 hypertensive patients were followed for 36 months. The incidence of stroke among hypertensive patients over the three year follow-up period was 106(18.18%) [95% CI 0.15–0.2], while 477(81.82%) [95% CI 0.78–0.85] were censored up to the end of the study. The overall incidence rate for diagnosed stroke patients registered at FHCSH during 11,208.26 person—year observations was 1 per 100 (95% CI 0.79–1.2) person year follow-up.

### Overall survival rate of stroke-free hypertensive patients

**Fig. 1:**
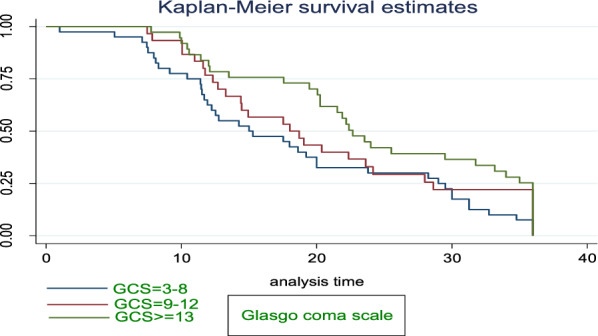
Kaplan–Meier survival curve based on admission Glasgow Coma Scale among stroke patients in FHCSH, during January 2018 to Feb2020, Bahir Dar, Ethiopia

The Kaplan–Meier survival estimate curve showed that the overall survival rate was 35.9% at the end of 36 months follow-up. The overall median survival time of stroke-free hypertensive patients were found to be 32.73 ± 9.0 IQR months (95% CI 31.3–36.0). Stroke patients with GCS 3–8 had 17.53 [95% CI 11.46–29.53] median survival times (Fig. 1).

### Survival estimate among predictor variables

**Fig. 2:**
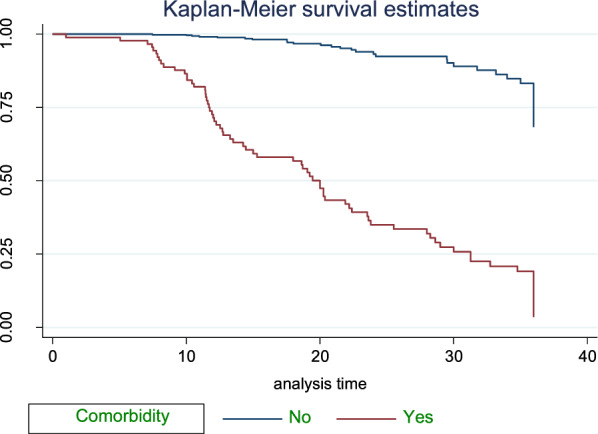
The KM survival function compare failure time of HTN patients with different categories of comorbid conditions in FHCSH, during January 2018 to Feb2020, Bahir Dar, Ethiopia

**Fig. 3:**
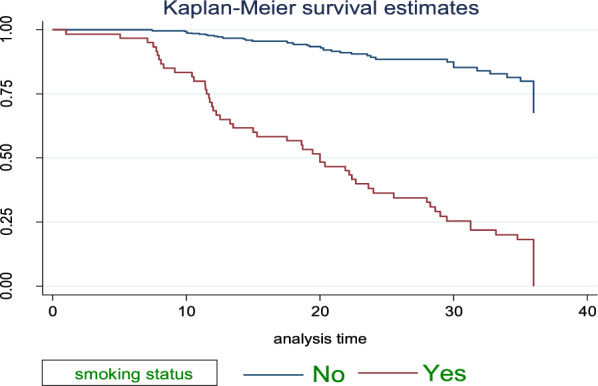
The KM survival function compare failure time of HTN patients with different categories of smoking status in FHCSH, during January 2018 to Feb2020, Bahir Dar, Ethiopia

**Fig. 4:**
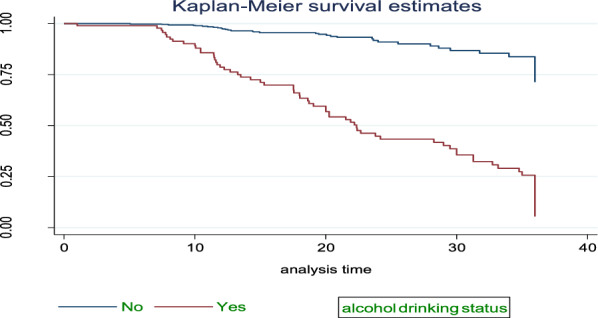
The KM survival function compare failure time of HTN patients with different categories of alcohol drinking status in FHCSH, during January 2018 to Feb2020, Bahir Dar, Ethiopia

**Fig. 5:**
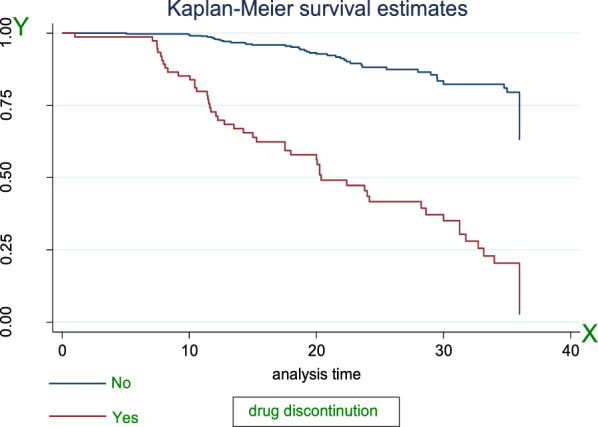
The KM survival function compares failure time of HTN patients with different categories of drug adherence status in FHCSH, January 2018 to Feb2020, Bahir Dar, Ethiopia

The Kaplan–Meier estimator survival curve provides the estimate of survivor function among different groups of covariates to make comparisons. Separate graphs of the estimates of the Kaplan–Meier survivor functions were constructed for different predictors. The test statistics (*p*-value ≤ 0.05) which is obtained from the log rank test clearly showed that there was a significant difference in survival curve for different categorical variables (Figs. 2, 3, 4, 5).

The study found that median survival time of hypertensive patients having comorbid conditions had lower survival than non-comorbid conditions 18.7 months as shown by statistical significance with *p*-value = 0.001. Patients with age from 45 up to 55 years had lower survival than others with median survival time of 31.27 at *p*-value = 0.001. Smokers and alcohol drinkers had lower survival than non-smokers and non-drinkers with median survival time of 13.5 and 19.07, respectively, at *p*-value = 0.001(Table3).

**Table 3:** Survival time and log-rank test for the study participants during three-year of follow-up of hypertensive patients in FHCSH, during January 2018 to Feb2020, Bahir Dar, Ethiopia (*N* = 583)

Variables	Categories	Median survival in month	Log-rank test (*p*-value)
Sex	Male	31.27	0.0013
	Female	32.7	
Age	< 45	32.89	0.001
	45–65	31.27	
	> 65	32.7	
Residence	Rural	31.77	1.001
	Urban	32.7	
Family history of stroke	Yes	7.77	0.001
	No	32.7	
Comorbidity	Yes	18.7	0.001
	No	33.17	
Smoking	Yes	13.5	0.001
	No		
Alcohol drink	Yes	19.1	0.001
	No		
Drug discontinuation	Yes	20.27	0.001
	No	34.8	
Level of SBP	< 140	33.2	0.001
	> 140	22.4	
Level of DBP	< 90	33.27	0.001
	> 90	22.3	
Follow-up frequency	Monthly	33.2	0.001
	Q2month	28.6	
	Q3month	11.9	

### Test of proportional hazard assumption

Testing the proportional hazard assumption is vital for interpretation and use of fitted proportional hazard models. Therefore, in this study goodness-of-fit (GOF) particularly the Schoenfeld residuals proportional hazard assumption test for the individual covariates and global tests was used. If *p*-value is below 0.05, then the proportional hazard assumption is rejected. From the table below, each covariate (*p*-value > 0.05) and all of covariates simultaneously (Global test for Cox proportional hazard *P*-Value =  > 0.05) met the proportional hazard assumption (Table 4).

**Table 4:** Goodness-of-fit test for assessing proportional hazards. Assumption of each covariates and overall survival model in FHCSH during January 2018 to 2020, Bahir Dar, Ethiopia

Predictors	Rho	chi2	D.f	Prob > chi2
Sex	− 0.033	0.15	1	0.7
Ethnicity	0.066	0.5	1	0.5
Residency	0.01	0.02	1	0.9
Family history	0.01	0.001	1	0.95
Q3 month frequency	− 0.101	1.39	1	0.24
Comorbidity	− 0.04	0.18	1	0.68
Smoking	− 0.05	0.30	1	0.58
Alcohol	0.017	0.04	1	0.84
Discontinuation	− 0.06	0.82	1	0.5
Age	− 0.08	0.67	1	0.41
Uncontrolled DBP	0.1	1.14	1	0.28
Uncontrolled SBP	0.033	0.15	1	0.7
Global test		7.15	19	0.99

### Factors associated with stroke among hypertensive patients

Both Bivariable and multivariable cox regression were done. After Bivariable cox proportional hazard regression done variables sex, age, follow-up frequency, comorbidity, smoking, alcohol consumption, drug discontinuation, and level of blood pressure were fitted at (*p* < 0.25). Those variables with *p*-value < 0.25 in the Bivariable analysis variables were included in multivariable analysis. In multivariable cox proportional hazards model age(45–65 years), alcohol consumption, comorbidity, follow-up frequency after three months, drug discontinuation and uncontrolled blood pressure above 140/90mmhg and stage two hypertension were significant predictors of stroke incidence (*p*-value < 0.05).

Hypertension patients in the age 45–65 years were 10.25 times at higher hazard of developing stroke (AHR: 10.25, 95%CI 7.47–11.1) than age below 45 years. Being female was 29% (AHR: 0.71, 95%CI 0.396–1.3) less likely to develop stroke compared with male patients.

Hypertensive patients having comorbid conditions were 1.8 times at higher hazard to develop stroke than patients with non-comorbid conditions (AHR: 1.8, 95%CI 1.0–3.5). Those hypertensive patients who smoke cigarettes’ and alcohol user were 1.46 verses 2.04 times at high hazard of developing stroke than non-smokers (AHR: 1.46, CI 0.77–2.77) non-alcohol users (AHR: 2.04, 95%CI 1.2–3.49), respectively.

Hypertensive patients who had uncontrolled systolic blood pressure were 2.17 times more likely to develop stroke than those having less than 140mmgh (AHR: 2.17, 95%CI 1.16–4.07) and those who had uncontrolled diastolic blood pressure were 2.14 times more likely to develop stroke than those who controlled their blood pressure (AHR: 2.14, 95%CI 1.2–3.77). Hypertensive patients who discontinued antihypertensive drugs were 2 times more at risk of developing stroke than patients who had good drug adherence (AHR: 2, 95%CI 1.26–3.34) (Table 5).

**Table 5:** Results of the Bivariable and multivariable cox regression of hypertensive patients in FHCSH, during January 2018 to Feb2020, Bahir Dar, Ethiopia. (*N* = 583)

Variable	Response	CHR 95%CI	AHR 95%CI
Sex	Male	1	1
	Female	0.55(0.36–0.84)	0.72(0.4–1.3)
Residency	Rural	1	1
	Urban	0.52(0.352–0.76)	0.98(0.6–1.58)
Family history	No	1	1
	yes	23.43(5.62–97.61)	2.78(0.95–8.13)
Comorbidity	No	1	1
	Yes	11.3(7.51–16.99)	1.88(1.0–3.54)^a^
Smoking	No	1	1
	Yes	8.53(5.8–12.53)	1.46(0.77–2.77)
Alcohol	No	1	1
	Yes	7.72(5.17–11.5)	2.04(1.2–3.49)^a^
Drug discontinuation	No	1	1
	Yes	7.04(4.81–10.32)	2.05(1.26–3.34)^a^
Age	< 45	1	1
	> 65	1.23(0.92–1.65)	10.25(7.47e + 08–2.1e + 09) 17.68
DBP	Controlled	1	1
	Uncontrolled	2.0(1.21–3.54)	2.0(1.21–3.54)^a^
SBP	Controlled	1	1
	Uncontrolled	1.9(1.1–3.57)	1.91.1–3.54^a^

^a^statistically significant at a given 95% CI *DBP* diastolic blood pressure, *SBP* Systolic blood pressure

## Discussion

In this retrospective follow-up study, 583 hypertensive patient cards were reviewed in the FHCSH medical ward and the result showed a high risk of hypertensive patients for stroke. The significantly associated predictors for the incidence of stroke among hypertensive patients were aged 45–65 years, alcohol consumption, comorbidities, drug non-adherence, uncontrolled systolic blood pressure, uncontrolled diastolic blood pressure, and follow-up frequency.

This study revealed that the incidence of stroke among hypertensive patients was found to be 18.18% [95% CI 15–20]. This finding is higher than the study conducted in Nigeria and Sidama with incidence of stroke 13.2% and 3.15%, respectively [26]. The discrepancy might be due to high prevalence of alcohol consumption and cigarette smoking. The incidence rate was 1 per 100 [95% CI 0.79–1.2] person-years. This finding is lower than a study conducted in Japan [46]. The difference could be due to follow-up time variation, absence of early screening and detection.

In this study, ischemic stroke is found to be 60 (56.6%) [95% CI 0.47–0.66] and haemorrhagic stroke covers 43.4% [95% CI 0.34–0.53]. This finding is persistent with the study conducted in Brno, Czech Republic [47]. The possible explanation might be due to the presence of various comorbidities like DM and other risk factors, which were pertinent attributes for ischemic stroke.

Hemiplegia/paresis was the most frequent clinical presentation in stroke patients with 99.05% followed by vomiting and decreased level of consciousness. This finding was in line with other studies [48, 49].

The higher percentage of stroke in male patients over females was in line with other previous studies [13, 50]. The possible reason may be increased risk factors such as cigarette smoking and alcohol consumption among males. In addition, there is no vascular protection of endogenous oestrogens in males.

Patients with age group 46–65 years were 10.25 times higher hazard of developing stroke than age under 45 years, and patients with age group > 65 years were 17.6 times had higher hazard of stroke than patients age group under 45 years. This Majority of stroke patients were in the age groups of 45–65 years (middle-adults) accounting 49.40% followed by older adults (> = 65 years) and younger adults [18–44 years] accounting 28.47% and 22.13%, respectively. These results were nearly similar with a study done in Jimma [50]. This was due to older people may have one or more cardiovascular diseases such as ischemic heart diseases, hypertensive heart diseases, high cholesterol, heart failure and the like as well as their sedentary lifestyles leads to older people have at greater risk of developing stroke.

In this study, hypertensive patients who discontinue anti-hypertensive drugs had two times higher hazard of stroke compared to others with who are not discontinue their drugs. This finding is in line with the study conducted in Tigray region, Ethiopia [38].

Patients with comorbidities had 1.8 times higher hazard of stroke than not having comorbidities. This is similar to the study conducted in China [51]. This study showed that 67.9% of stroke patients have different comorbidities. Among this, Diabetes mellitus cover 33.3%.

This could be because of diabetes, which can lead to pathological changes in blood vessels at various sites, particularly if cerebral vessels are directly affected. Another possibility is that if there is too much glucose in the blood, fatty deposits or blood clots form on the inside of blood vessels, which can narrow or block the blood vessels in the brain, preventing oxygen from reaching the brain and resulting in stroke.

Patients who had followed up more than 3 months had 2.7 times higher hazard of stroke compared with others followed up monthly This finding is similar to the study conducted in Tigray region [38]. This similarity might be due to missing their routine medications and lifestyle modification counselling leads to uncontrolled hypertension.

Alcohol drinkers had two times higher hazard of stroke compared to non-drinkers. This finding was in line with the study conducted in Tigray region, Ethiopia [38]. This similarity might be due to alcohol having a direct impact on raising blood pressure.

Smokers had 1.46 times higher hazard of stroke compared to non-smokers. This finding is similar to the study conducted in Jimma, Ethiopia [50]. The possible explanation might be due to smoking may predispose blood vessels to thrombosis and facilitate platelets aggregation possibly by causing an imbalance between brain vascular coagulation and abnormal fibrinolysis. This might alter the function of the blood brain barrier and disrupt normal endothelial cell function.

Uncontrolled systolic blood pressure was 2.0 times more hazards for the development of stroke and uncontrolled diastolic blood pressure were 1.9 times more hazards for the development of stroke. This is in line with the study conducted in Paget [52]. This is due to uncontrolled blood pressure increases the pressure of blood flow through the arteries as a result of this leads to damaging and weakening of blood vessels causing them too narrow, rupture or leak and also causes blood clots to form in the arteries leading to the brain, blocking blood flow and causes a stroke. This study has the following potential limitations: This is a hospital-based study and as such the results cannot be generalized to the general population. Selection bias might possibly be introduced during secondary data collection because patients with incomplete records were excluded so that the incidence of stroke may be under or over estimated. Loss to follow-up might be biased to the incidence of stroke among hypertensive patients. Since the data collected from secondary source; some important predictors such as biological biomarkers, treatment adherence, physical exercise, BMI, educational status, marital status and multidisciplinary care were missed which might have significant predictions for incidence of stroke.

## Conclusion

In this study, higher incidence rate of stroke among hypertensive patients in Felege Hiwot Specialized Hospital was identified. Age (45–65 years), alcohol consumption, comorbidity, follow-up frequency after three month, drug discontinuation, and uncontrolled blood pressure found to be significant predictors for the incidence of stroke among hypertensive patients. Therefore, we recommend early screening of blood pressure, giving priority to comorbid patients and patients with advanced stage hypertension, and giving health education about behavioural risks and drug adherence.

## Data Availability

The necessary data sets used during the current study are available from the corresponding author on reasonable request.
